# Shaping suvorexant: application of experimental and theoretical methods for driving synthetic designs

**DOI:** 10.1007/s10822-014-9710-x

**Published:** 2014-02-01

**Authors:** Georgia McGaughey, Christopher I. Bayly, Christopher D. Cox, John D. Schreier, Michael J. Breslin, Michael Bogusky, Steve Pitzenberger, Richard Ball, Paul J. Coleman

**Affiliations:** 1 Chemistry, Modeling and Informatics, Merck Research Laboratories, WP53F-301, West Point, PA 19486 USA; 2 OpenEye Scientific Software, Santa Fe, NM 87508 USA; 3 Discovery Chemistry, Merck Research Laboratories, WP14-3, West Point, PA 19486 USA; 4 Analytical Chemistry, Merck Research Laboratories, Rahway, NJ 07065 USA; 5 Present Address: Vertex Pharmaceuticals, 50 Northern Ave, Boston, MA 02210 USA

**Keywords:** Suvorexant, Conformational analysis, Free energy, ROCS

## Abstract

Dual Orexin Receptor Antagonists (DORA) bind to both the Orexin 1 and 2 receptors. High resolution crystal structures of the Orexin 1 and 2 receptors, both class A GPCRs, were not available at the time of this study, and thus, ligand-based analyses were invoked and successfully applied to the design of DORAs. Computational analysis, ligand based superposition, unbound small-molecule X-ray crystal structures and NMR analysis were utilized to understand the conformational preferences of key DORAs and excellent agreement between these orthogonal approaches was seen in the majority of compounds examined. The predominantly face-to-face (F2F) interaction observed between the distal aromatic rings was the core 3D shape motif in our design principle and was used in the development of compounds. A notable exception, however, was seen between computation and experiment for suvorexant where the molecule exhibits an extended conformation in the unbound small-molecule X-ray structure. Even taking into account solvation effects explicitly in our calculations, we nevertheless find support that the F2F conformation is the bioactive conformation. Using a dominant states approximation for the partition function, we made a comprehensive assessment of the free energies required to adopt both an extended and a F2F conformation of a number of DORAs. Interestingly, we find that only a F2F conformation is consistent with the activities reported.

**Electronic supplementary material:**

The online version of this article (doi:10.1007/s10822-014-9710-x) contains supplementary material, which is available to authorized users.

## Introduction

Orexins, or hypocretins, are neuropeptide hormones that have been shown to regulate arousal and wakefulness [1]. There remains debate in the scientific community as to whether these neuropeptides should be referred to as orexins or as hypocretins after being simultaneously discovered by two independent research groups [2, 3]. The term orexin originates from the Greek word, orexis, which means appetite, while hypocretin is derived from the observation that it is secreted in the lateral hypothalamus and is similar to the hormone secretin. Herein, we will refer to the neuropeptides as orexins and the compounds that antagonize both the Orexin 1 and 2 receptors (OX1R and OX2R, respectively) as Dual Orexin Receptor Antagonists, or DORAs.

Orexinergic neurons project to different areas in the central nervous system, which include areas of the brain that regulate the sleep-wake cycle, and there is strong genetic and pharmacological evidence implicating the role of orexins in sleep-wake regulation. Indeed, blockade of orexin signaling by small-molecule antagonists has been shown to promote sleep in preclinical species and in human clinical trials [4].

In previous publications from this laboratory we described the development of numerous chemotypes (diazepanes, tetrahydroisoquinolines, diazaspirodecanses and proline amides) arising from a successful high-throughput screening campaign [5]. For the purposes of this manuscript, we focused only on the diazepane ring structures. We will describe the 3D shape motif, conformational analysis, and X-ray structure of suvorexant.

## Discovery and exploitation of core 3D shape motif

**Fig. 1:**
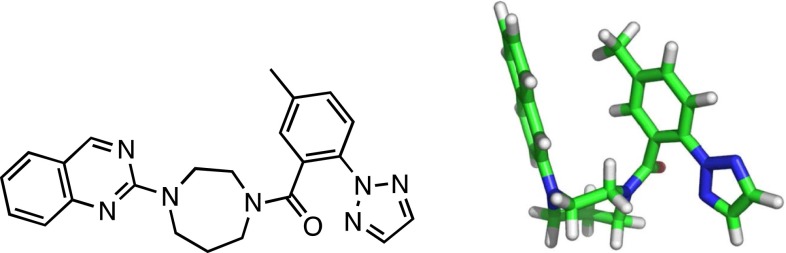
2D and 3D small molecule low energy, computationally-derived representation of Compound **1** (OX1R Ki = 1.2 nM, OX2R Ki = 0.8 nM. Individual replicates for all data shown herein are included in the supplementary material. Only the mean is reported

As previously described [6], a strong correlation between the predicted bioactive conformation of compound **1** (Fig. 1) and experimental methods was established.

Initially, we used the mixed torsion/low-mode sampling algorithm implemented in Maestro [7]^1^ with the OPLS 2005 force field in the gas phase which suggested a folded, face-to-face (F2F) conformation wherein distal ring systems pack against each other as depicted in Fig. 1. Given that dispersion forces can sometimes be too large in force-fields which might lead to a bias towards F2F conformations, we invoked additional computational methods (e.g. quantum mechanical), included solvent to aid or refute the effects of hydrophobic collapse and experimented with using different force fields (Table 1).

**Table 1:** Effect of solvent and level of theory on the predominant conformation of compound **1**

Compound **1**	F2F^a^	Extended^a^
OPLS 2005 gas	0.0	2.7
OPLS 2005 solvent	0.0	1.1
OPLS gas 2.1	0.0	3.6
OPLS water 2.1	0.0	2.7
MMFFs gas	0.0	1.1
MMFFs solvent	0.0	2.1
RHF/6-31G** gas	0.0	1.4
RHF/6-31G** PBF	0.0	2.9

^a^All values reported in kcal/mol and the minimum value is normalized to zero

The results of this study demonstrated that there was excellent agreement between the molecular mechanics force fields, MMFFs and OPLS and the use of solvent did not affect the predominant conformation. The use of solvent in the quantum mechanical calculations was not required to achieve the F2F conformation as the hypothesized bioactive conformation. All reported conformations were minimized to convergence [7].

Although the molecular and quantum mechanical methods were in agreement with one another, we sought additional experimental methods to either support or refute our initial computational findings. We first determined the unbound, small-molecule crystal structure. The RMSD between the computationally derived conformation depicted in Fig. 1 and the X-ray structure was 0.75 Å (non-hydrogen atoms). Additionally, no differences between the geometries of the nitrogen atoms of the seven-membered ring nor the amide bond were observed between the theoretically and experimentally derived conformations. Even though there was no computed difference between the conformations (Table 1) when implicit solvent was invoked, we sought to experimentally confirm that the solution state conformation was consistent with the aforementioned theoretical and crystal structure experiments. There was again excellent agreement from NMR spectroscopy applied to compound **1**, with NOE evidence for the F2F interaction in the major rotamer. In fact, two F2F rotamers in the ensemble accounted for 82 % of the total solution conformation (CD_3_OD at −40 °C), each with a motif similar to that found by computational analysis and X-ray structure.

We were encouraged that the solid-state, solution and theoretical data all correlated and so further tested the importance of the F2F interaction through synthetic design. If the F2F motif was the predominant active conformation, then we hypothesized that a macrocyclic structure which forced such a motif to exist should be at least equally active, within experimental error. Indeed, as was previously published, this hypothesis was confirmed [6]. As predicted, the OX1R and OX2R binding values of the synthetic precursor and macrocycle DORAs were within experimental error (Table 2).

**Table 2:**
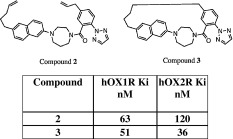
An acyclic (compound **2**) and cyclic (compound **3**) structure was synthesized and tested in both the Orexin 1 and Orexin 2 receptor binding assay

We were confident at this juncture that the 3D motif was reflective of the bioactive conformation. We had (1) two experimental methods (unbound small-molecule X-ray and NMR) corroborating our conformational analysis and (2) similar biological results attained from both the acyclic and macrocycle compounds. Thus, the F2F interaction became an important criteria adopted in our synthetic work, and we analyzed compounds based on whether they could attain this unique 3D shape motif.

## Quantification of F2F using ROCS

Even though the orexin 1 and 2 targets were known and we did generate homology models of the receptors, we found the ligand based drug design approach to be more successful. We used a combination of Merck’s proprietary method, SQW [8] and OpenEye’s method ROCS [9], both 3D shape methods, to rank order the ability of compounds **4**–**7** [10, 11] to conform to our proposed bioactive form, the F2F motif. Both these tools are ligand shape-based methods and we utilized them to analyze compounds. For this manuscript, however, we will report the ROCS Tanimoto Combo Score values as they can be independently reproduced.

Once we had experimental confirmation that compound **1** did indeed adopt a F2F conformation, we used the small-molecule X-ray conformation as our reference and calculated: (1) the low energy conformations using a combination of the MMFFs and/or OPLS force field [7], and (2) the Tanimoto Combo score (a sum of shape and 3D chemical Tanimoto values) as implemented in ROCS (Table 3), [9]. Initially, we generated conformations as previously published [6] but as we progressed into lead optimization, we expanded our conformational sampling by using OMEGA and increased the energy range and number of conformations to ensure larger conformational sampling [12]. Thus, the Tanimoto Combo scores reported in Table 3 are a result from assessing large ensembles of conformations (>100) and reporting the best (highest) Tanimoto Combo score.

**Table 3:** ECFP4 and Tanimoto scores relative to the 2D and crystal structure of compound **1**

Compound	OX1R^a^	OX2R^a^	ECFP4 similarity^b^	ROCS Tanimoto Combo Score^b^	ROCS Tanimoto Combo Score no rings^b^
**1**	1.2	0.8	1.000	1.722	1.575
**4**	0.4	0.6	0.727	1.146	1.242
**5**	>1,700	2740	0.717	0.928	0.955
**6**	0.6	0.2	0.731	1.618	1.499
7*	0.54	0.35	0.667	1.622	1.587

* Compound **7** is also known as MK-4305 or Suvorexant

^a^OX1R and OX2R Ki values are reported as nM

^b^ECFP4 and Tanimoto scores of each compound relative to the small molecule X-ray structure of compound **1**

As expected, compound **1** exhibited a high Tanimoto Combo score since it was calculated relative to itself, thus tests our facilities to computationally find this presumably low energy conformation. As can be seen in Table 3, all compounds except compound **5** exhibit ROCS Tanimoto Combo scores suggested of activity based on a publication by Muchmore et al. [13]. In contrast, compound **5** has a lower ROCS Tanimoto Combo score giving us further confidence that the F2F shape motif discriminates between actives and in-actives. Compound **4** exhibits a ROCS Tanimoto Combo score in the low predicted activity range; however, it is still higher relative to Compound **5**.

It is known that ROCS can in some cases, overcompensate for initial overlaps based on the chemical nature of existing rings in the ligands. The score for compound **5** is such that the Muchmore scale would not have anticipated activity. We wondered, however, if the reason compound **4** was not more highly ranked was the default behavior of the ROCS scoring function towards rings, i.e. whether the bridged rings in compound **4** and **5** were not being ‘rewarded’. The last column in Table 3, wherein we use the “No Rings” version of the ROCS overlap function, suggests this was indeed the case. In general scores are expected to be lower and were, however compound **4**, and compound **5** to a lesser degree, improved. Compound **5** would still be classified as likely inactive, whereas all the other compounds, including now compound **4**, would be solidly classified as likely active.

We did attempt to correlate strain energy to biological activity but did not find this a good metric for distinguishing actives from inactives as the accessible conformations were all relatively low strain. These strain energy calculations did not take into account entropy. It is worthwhile to note that the topological method, ECFP4 [14], was not able to distinguish actives from inactives and further underscore the importance of using 3D shape in this study.

**Fig. 2:**
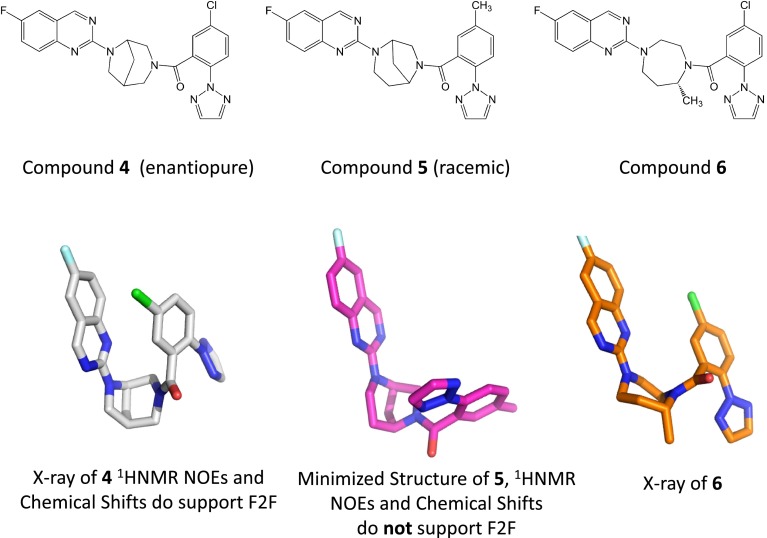
2D representation of chemical structures and corresponding 3D low energy conformation of ligands

We analyzed compounds **4**–**7** using the ROCS and SQW methodologies which are designed to virtually screen molecules based on shape. In the case of compound **4**, both X-ray and NMR structures could be determined and it was established that indeed, the F2F conformation was predominant (Fig. 2). In contrast, compound **5** was shown by conformational analysis to not present itself in the F2F conformation and turned out to be >1,000 times less active than compound **4**. The lack of predicted F2F activity was supported experimentally by the absence of observed NOE correlations between the distal aromatic rings. Compound **6** was predicted to present the distal aromatic rings in a F2F arrangement and this was experimentally observed in the small-molecule X-ray structure. In all three cases (compounds **4**–**6**), the computational analysis was supported by experimental methods and by a biological binding assay. These results are summarized in Fig. 2.

**Fig. 3:**
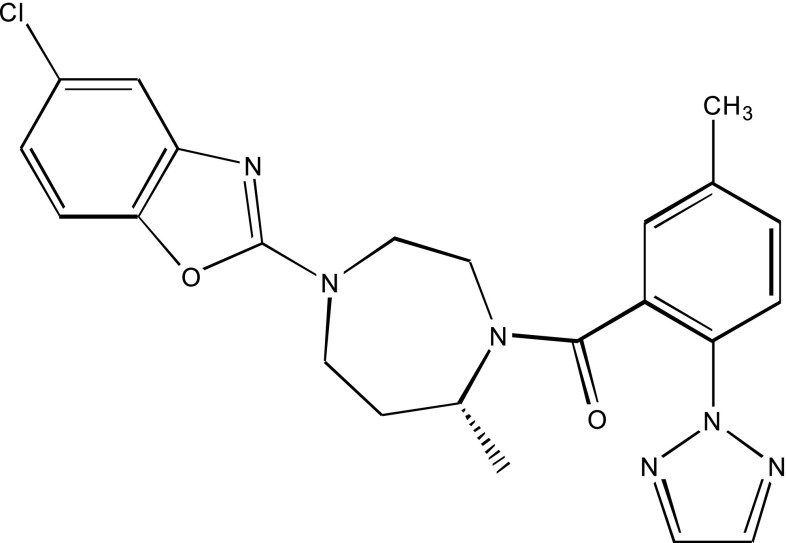
2D representation of compound **7** which became our clinical candidate (MK-4305) and later termed Suvorexant (OX1R Ki = 0.54 nM, OX2R Ki = 0.35 nM)

Compound **6** demonstrated excellent in vivo activity but was subsequently found to exhibit a metabolic liability [10]. A 6,5 heterocycle, the benzoxazole, was unexpectedly found to be a replacement for the quinazoline ring that is devoid of the metabolic issues in compound **6** (compound **7**, Fig. 3) [15]. Since the only change was the bicyclic heterocycle, the ROCS Tanimoto Combo Score for Compound **7** of 1.622 was similar to that of compound **6** (1.618). Compound **7** was studied in vivo and shown to promote sleep in preclinical species in a dose dependent fashion and also demonstrated a favorable toxicology profile.

Further analysis concluded that compound **7** was highly selective for orexin receptor binding and the compound was CNS penetrant with a rat brain/plasma ratio of 0.6–1.2 in an *iv* infusion study. Compound **7** (MK-4305) was advanced as a clinical candidate and was termed suvorexant.

## Is the 3D motif “true”?

**Fig. 4:**
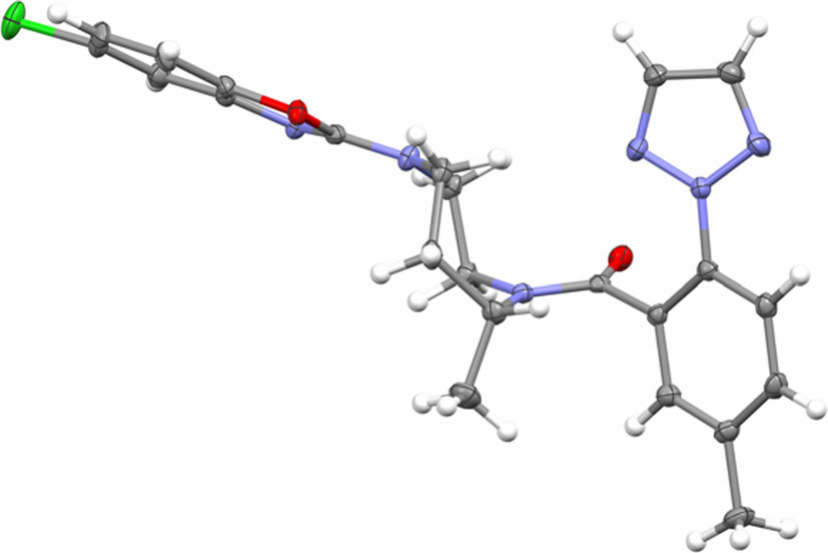
X-Ray structure of Suvorexant (MK-4305/Compound **7)**

In parallel to our back-up efforts, suvorexant continued to progress through all phases of clinical trials (currently in Phase III). As part of this process we obtained an unbound small molecule crystal structure of suvorexant and were surprised to observe that the molecule adopts an *extended* conformation crystallographically, not the predicted F2F arrangement (Fig. 4). Our first reaction was to postulate that perhaps one of the amines on the ligand could be protonated and that may have altered the ability to adopt a F2F conformation. We subsequently grew crystals from a supersaturated (55 °C) ethyl acetate solution to test this hypothesis. This second X-ray structure still exhibited an extended conformation. Our next option was to apply NMR spectroscopy since we had previously shown a strong correlation between these methods (i.e. computational chemistry, X-ray crystallography and NMR spectroscopy). Again, we presumed that a solution-state conformation may be more biologically relevant than the corresponding solid-state conformation seen in the X-ray structure. In two NMR studies, (CD_2_Cl_2_ and CD_3_OD), there was no evidence of a predominant F2F interaction.

Given the earlier agreement of experimental and theoretical methods as to the dominance of the F2F motif in solution (and crystal forms) we did not anticipate this finding especially since the data for the macrocycle (compound **3**) supported our hypothesis of the F2F motif. Our fall-back assumption had to be that in modifying earlier compounds the preferred conformation in solution shifted from F2F to extended, but that to continue to be efficacious the bound molecule reverted to the F2F form. Without a co-crystal structure of suvorexant bound to OX1R or OX2R we cannot substantiate this hypothesis. However, this would be unlikely if the energy difference between these two forms in solution was large. As such we examined the energetics of each in some detail (Table 4). At the time of writing this manuscript a new version of OPLS became available (OPLS 2.1) and we found that results obtained with this newer version were in agreement with the quantum mechanical studies whereas OPLS 2005 was not.

**Table 4:** Energetic differences between the extended and F2F shape motif of Suvorexant using different levels of theory

Compound **7**	F2F^a^	Extended^a^
OPLS (2005) gas	0.0	1.1
OPLS (2005) solvent	0.0	1.1
OPLS (2.1) gas	1.1	0.0
OPLS (2.1) solvent	0.15	0.0
MMFFs gas	1.4	0.0
MMFFs solvent	0.62	0.0
RHF/6-31G** gas	2.7	0.0
RHF/6-31G** PBF	1.7	0.0

^a^Energies are in kcal/mol and the lowest energy structure is normalized to zero

It is readily apparent that the OPLS 2005 method, which was used in our original diazepane designs, identified the extended conformation within 1.1 kcal/mol of the global minimum. However, the extended conformation was predominant when the MMFFs and OPLS 2.1 force fields were utilized and continued to be predominant when implicit solvent was included in the calculation. Coincidentally, both quantum mechanical methods RHF/6-31G** and the MMFFs and OPLS 2.1 methods were in agreement. Irrespective, the energetic differences between the F2F and extended conformations are quite modest suggesting that multiple conformations of suvorexant are energetically possible.

**Fig. 5:**
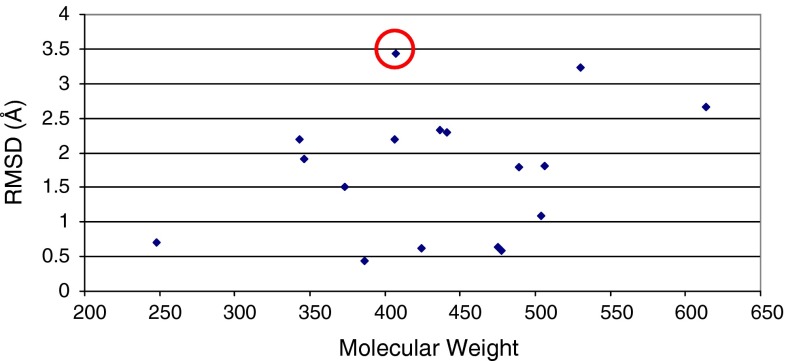
RMSD between the non-hydrogen atoms of the same compound in the bound (complex) and unbound (no complex) crystal structure as a function of molecular weight. The value for Januvia^®^ is circled in red

Although numerous computational tools, theories and concepts can be utilized to rationalize an experimental result, it is typically most satisfying to have experimental data to support an outcome. Thus, we turned our analysis to Merck’s in-house small-molecule and complexed crystallographic data to help understand the differing suvorexant results. We searched for small molecules where crystal structures of both the unbound and bound forms were experimentally determined. We then calculated the RMSD between the non-hydrogen atoms of the ligand in both structures. To ensure we were not biasing towards molecules with low molecular weight (and thus, likely to have relatively fewer rotatable bonds), we examined 17 compounds from our in-house crystallography database over a range of molecular weight values. As is demonstrated in Fig. 5, there is often a difference between the bound and unbound ligand conformations. In fact, Merck’s drug for the treatment of diabetes (Januvia^®^) shows a marked difference between the bound and unbound crystal structures (Fig. 5) [16].

**Fig. 6:**
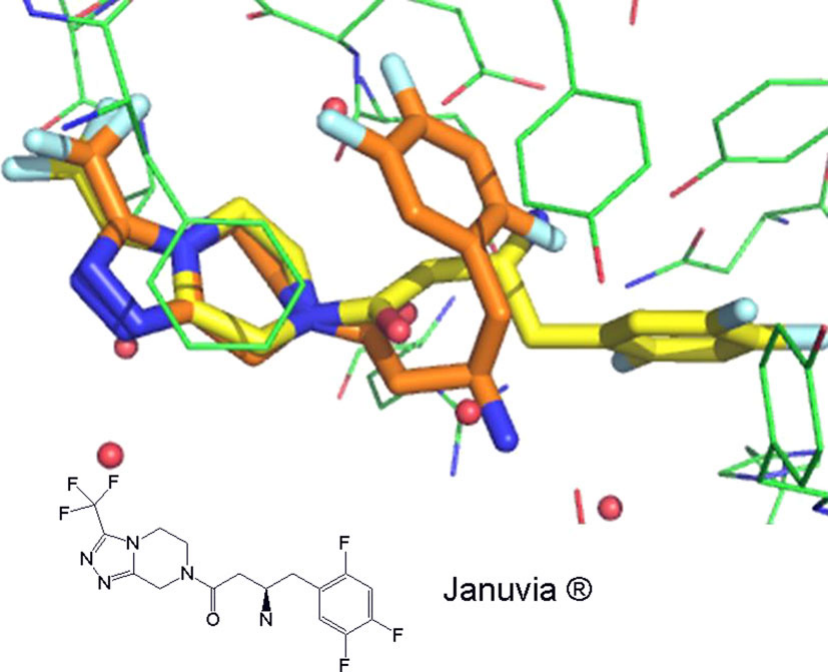
Small-molecule crystal structure colored in orange (carbon atoms) of Januvia^®^ superposed with bound crystal structure of Januvia^®^ colored in yellow (carbon atoms) complexed in DPPIV

The literature also suggests that while the predicted low energy solvated structure is often the bioactive this is not always true [17, 18] (Fig. 6).

Up to this point, we had developed our F2F hypothesis based on the solvation-corrected force field *relative* energies, assuming that these enthalpic contributions to the overall bioactive conformation would approximate the free energy of binding (i.e. ΔH = ΔG), excluding any entropic contributions. We had completely omitted the possibility that perhaps the free energy would be more indicative of the solution state conformation than examination of the relative energy. As a result, we sought to incorporate the vibrational and rotational entropic contributions to the free energy using implicit solvent.

## Relative energies versus free energies

Conformer free energies, defined here to mean the energy required to select one conformer from the ensemble of all conformers in solution, were computed for compounds **1** and **7**. A high-resolution conformer database was generated using OMEGA [12] followed by energy minimization (including Sheffield solvation [19]), followed by Poisson-Boltzmann single-points for improved solvation estimates. Conformer free energies were computed based on the partition function *Q* summed over conformer partition functions *Q*_*i*_:

1 $$Q = \sum\nolimits_{i} {Q_{i} }$$

where:

2 $$Q_{i} = q_{iv} q_{ir} e^{{ - E_{rel}^{i} /RT}}$$

and for each conformer *i*:

3 $$E_{rel} = E_{FF} + E_{solv} - E_{min}$$

The vibrational partition functions *q*_*v*_ were calculated using analytic second derivatives (including Sheffield solvation) at each minimum [19]; the rotational partition function *q*_*r*_ were calculated based on moments of inertia. Translational entropy was ignored because it cancels between conformers. For each minimized conformer the force field energy *E*_*FF*_ and the solvation energy *E*_*solv*_ are summed and the global energy minimum *E*_*min*_ is subtracted to yield the relative energy. The conformer free energy for conformer *i* is then given by:

4 $$\varDelta G_{i} = - RT(\ln (Q_{i} ) - \ln (Q))$$

**Fig. 7:**
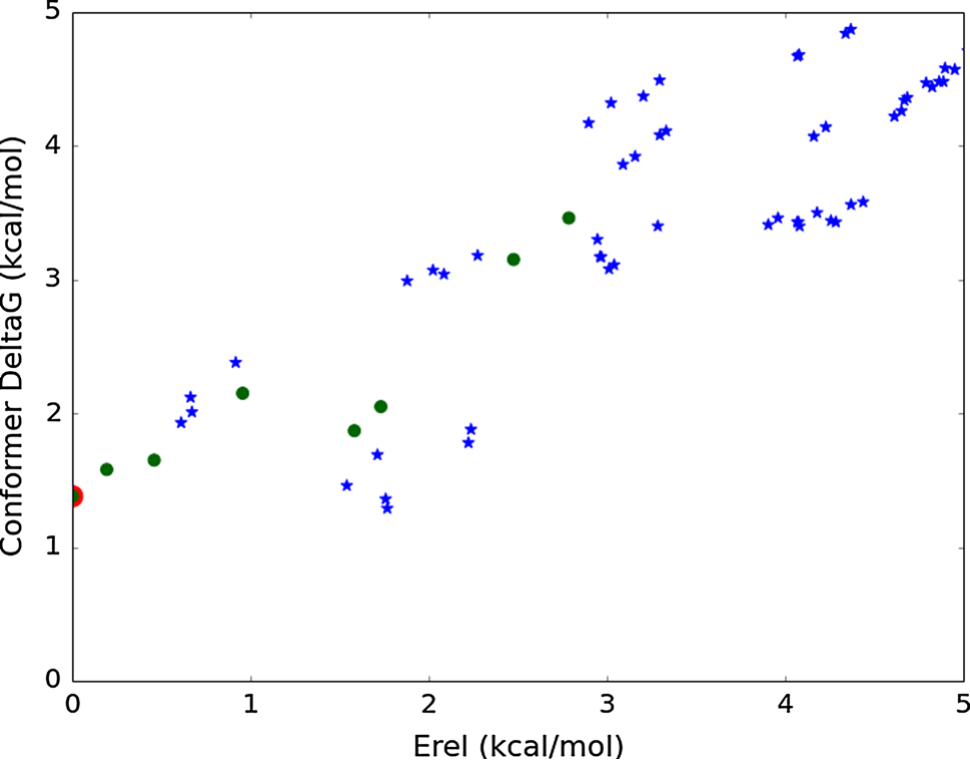
Conformer free energies versus relative energies (force field + solvation) for accessible conformations of **1**. *Blue stars* Extended conformations; *green circles* F2F conformations. The model for the bioactive conformation (F2F) is outlined in *red*. There are extended conformers close in free energy

Figure 7 shows that the conformer free energies for **1** differ substantially from the relative energies; while the global relative energy minimum conformation (F2F) shown in Fig. 1 has one of the lowest conformer free energies, there are extended conformers with comparably low free energies. Allowing for inaccuracies in the force field and for the solvation differences between water and CD_3_OD, we think this is consistent with the 82 % prevalence of F2F conformers in the NMR experiment mentioned earlier.

**Fig. 8:**
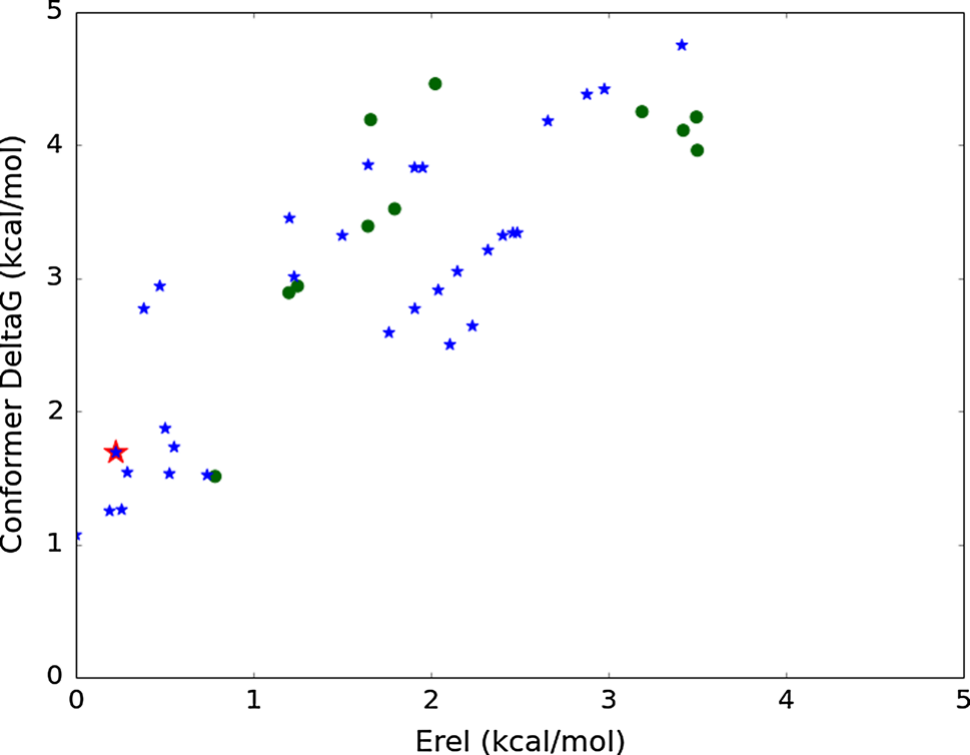
Conformer free energies versus relative energies (force field and solvation) for accessible conformations of **7**. *Blue stars* Extended conformations; *green circles* F2F conformations. The minimum corresponding to the X-ray conformation (extended) is outlined in *red*

Compound **7** exhibits a markedly different free energy distribution of F2F versus extended conformers as shown in Fig. 8. Now most of the lowest free energy conformers are all extended, with the crystal structure conformer among them. This also explains why the solution NMR structure reflects an extended conformation. Nevertheless, a F2F conformer is still found amongst this cluster having the lowest free energy and other F2F conformers are found a couple of kcal/mol higher in free energy. Thus it is apparent that for compound **7**, while the F2F conformations postulated for biological activity are not prevalent, they are nevertheless energetically accessible. The free energy cost to adopt the F2F conformation is low and so could be easily compensated for by binding free energy.

## Rationalizing suvorexant’s 3D motif

Although we don’t yet have the unequivocal answer for how suvorexant binds, the differing results between theory and experiment can be rationalized as follows: (1) the small molecule X-ray structure could be influenced by crystal packing forces (2) the NMR conformations could be a result of solvent effects (3) the conformational analysis results invoking different levels of theory suggest that there are low lying energy populations readily accessible (4) an analysis comparing the bound and unbound ligand forms indicates that the small molecule X-ray structure does not always correlate with the bound conformation or (5) the simplest rationale: that we need to determine the free energy of the compound in solution through the explicit determination of the vibrational and rotational entropic contributions.

While we are hopeful that advances in GPCR crystallography will come to shed light on this puzzling difference, we believe this work presents a cautionary tale for ligand-based design. If we had been unfortunate to have started with an active molecule similar in conformation to the unbound X-ray structure of suvorexant, we might not have recognized the F2F motif. That said, we would have likely challenged the F2F motif to be a possible bioactive conformation since the F2F conformation is energetically quite accessible as determined by multiple computational methods. It was the application of *multiple* *orthogonal* *methods* incorporating experimental tools that aided in developing a testable hypothesis.

## Conclusion

In the work presented here we follow a tight hypothesis: that a predominantly F2F motif is the bioactive conformation for active DORAs. Whether the structure of suvorexant bound to OX1R and/or OX2R turns out to be extended or not, we were successful in applying our F2F motif hypothesis, derived from a thorough understanding of the shape of the ligand. The important aspect of this approach was relying on *multiple* *orthogonal* *methods* to investigate the bioactive conformation. Clearly, in the quest for a novel sleep medication, the integration of many experimental and theoretical methods “worked” to refine the hypothesis and shape based analysis helped to develop Merck’s DORAs.

In trying to understand the experimentally determined preference for compound **7**, we found that a critical role was played by methyl substitution on the seven membered diazepane ring. While this introduced a marginal preference for the extended conformation in some cases (Fig. 8), the F2F conformation was always within a kcal/mol of the global minimum and often preferred.^2^

The use of the partition function ensures that we have incorporated all the important energetically accessible energy minima including both the F2F and extended conformations. Secondly, by including the vibrational and rotational entropic contributions of each conformer, we find that these influence whether the F2F or extended conformation are preferred within a force field—or within the same force field potential energy surface. Irrespective, the energy differences were within a 1 kcal/mol lending us to conclude that both F2F and extended conformations are significant contributors to the unbound conformational ensemble in solvent.

## Electronic supplementary material

Below is the link to the electronic supplementary material.

Supplementary material 1 (XLSX 12 kb)

## Footnotes

1 Conformational searching using the mixed torsion/low-mode sampling algorithm as implemented in Schrödinger, v9.0 (Schrödinger, LLC, v9.0) was utilized with default values. It was found that the number of maximum iterations using the PRCG method needed to be increased to 5,000 for convergence to occur.

2 When combined with other structural modifications, incorporation of the 7-methyl substituent on the diazepane ring was discovered to have an unexpectedly favorable effect on the pharmacokinetic profile. See Ref. [10].
